# Multiple-target Therapy for Posttransplant Focal Segmental Glomerulosclerosis

**DOI:** 10.1097/TXD.0000000000001651

**Published:** 2024-05-28

**Authors:** Juliana Mansur, Domingo Chang-Dávila, Marcela Giraldes Simões, Marina Pontello Cristelli, Suelen Bianca Stopa Martins, Henrique Machado de Sousa Proença, Laila Almeida Viana, Alexandra Nicolau Ferreira, Marisa Petrucelli Doher, José Medina-Pestana, Gianna Mastroianni Kirsztajn, Helio Tedesco-Silva

**Affiliations:** 1 Nephrology Division, Hospital do Rim, Fundação Oswaldo Ramos, São Paulo, Brazil.; 2 Nephrology Division, Universidade Federal de São Paulo (UNIFESP), São Paulo, SP, Brazil.; 3 Universidade Peruana Cayetano Heredia, Lima, Peru.

## Abstract

**Background.:**

There is no consensus on the ideal strategy to treat posttransplant focal segmental glomerulosclerosis. The multiple-target therapy, which consisted of high-dose intravenous cyclosporine, prednisone, and plasmapheresis, showed favorable results.

**Methods.:**

This single-center, prospective study sought to evaluate the multiple-target therapy in an independent cohort of patients.

**Results.:**

Thirteen patients with posttransplant focal segmental glomerulosclerosis received multiple-target therapy. Complete remission was achieved in 2 patients (15.4%), and partial remission in another 2 patients (15.4%). Four patients (30.7%) did not show remission, and 5 patients (38%) lost the graft because of posttransplant focal segmental glomerulosclerosis during the 12-mo follow-up. Premature discontinuation of treatment occurred in 10 patients (77%), all associated with infectious adverse events. Cytomegalovirus was the most common complication, and preemptive therapy was used instead of prophylaxis.

**Conclusions.:**

In this cohort of patients, the efficacy of the multiple-target therapy was poor and limited by the high incidence of infectious adverse events.

## Graphical abstract

**Figure:**
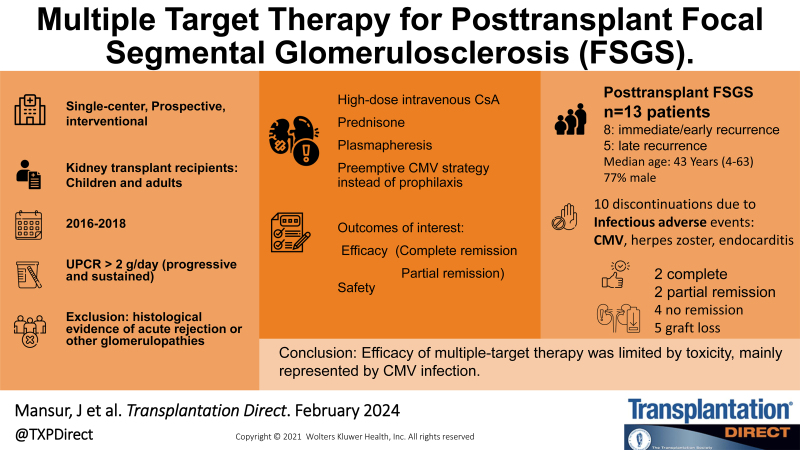

The recurrence of focal and segmental glomerulosclerosis (FSGS) occurs in 30% to 80% of patients after kidney transplantation (KT), predominantly occurring within the initial 6 mo posttransplantation. This phenomenon is likely attributable to podocyte injury induced by a circulating permeability factor.^1-4^ Conversely, de novo FSGS, whether primary or secondary, tends to manifest at a later stage, typically beyond the first year after transplantation.^5^

Despite its association with an increased risk of graft loss,^6^ there is still no consensus concerning the optimal therapeutic strategy for posttransplant FSGS. Retrospective investigations have revealed a wide range of remission rates (20%–80%) and high rates of relapse and/or plasmapheresis (PP) dependency in both adults and children treated solely with PP. Studies using a combination of rituximab and PP have reported inconsistent efficacy, albeit with an acceptable safety profile.^7^ In a retrospective study including 61 KT recipients (KTRs) with posttransplant FSGS, the findings revealed a 30% rate of complete remission (CR) and a 20% rate of partial remission (PR) when treated solely with PP. Notably, this response rate did not exhibit improvement with the addition of high-dose steroids.^8^

In 2009, in a nonrandomized pilot trial, Canaud et al^9^ reported that the use of a “multiple-target therapy,” consisting of high-dose intravenous cyclosporine (CsA), prednisone, and PP for a period of 9 mo, achieved CR in 90% of the KTRs with a favorable safety profile. This study sought to confirm the efficacy and safety of this therapeutic strategy using an independent cohort of patients.

## MATERIALS AND METHODS

### Study Design and Population

This was a single-center, prospective, interventional, noncontrolled study investigating the efficacy and safety of multiple-target therapy for posttransplant FSGS. The study was approved by the local Ethics Committee and all patients signed an informed consent form.

The population of interest consisted of KTRs of all ages who, during the period of April 20, 2016, to July 11, 2017, had a clinical suspicion of FSGS, defined as progressive and sustained proteinuria >2 g/d after transplantation. Follow-up of patients continued until June 2018 or until the date of allograft loss or recipient death. Based on the availability of a native kidney biopsy, patients were classified into “confirmed FSGS” or “presumed FSGS.” All patients were submitted to a kidney allograft biopsy and a laboratory workout to exclude other diagnoses. Patients were excluded from analysis if there was any histological evidence of acute rejection or other glomerulopathies.

### Multiple-target Therapy for FSGS Recurrence

Patients received the multiple-target therapy comprising of high-dose intravenous CsA, prednisone, and PP after 3 d of permanent nephrotic range proteinuria without spontaneous improvement.^9^ The initial 2 mg/kg intravenous dose of CsA was adjusted to maintain whole blood trough CsA concentrations between 200 and 400 ng/mL for 14 d, transitioned to an initial 3–5 mg/kg oral dosing, targeting similar blood concentrations. Patients also received 1 mg/kg/d of prednisone during the first 4 wk, tapered to 0.75 mg/kg/d for 2 wk, followed by 0.5 mg/kg/d for 2 wk, 0.25 mg/kg/d for 2 wk, and 0.2 mg/kg/d or a maximal daily dose of 10 mg thereafter. Finally, PP with 5% albumin replacement was started at 3 sessions per week for 3 wk, followed by 2 sessions per week for 3 wk, 1 session per week until month 3, 2 sessions per month until month 5, and, finally, once a month until month 9.

### Prophylaxis Against Infectious Complications

All patients received a 5-d course of albendazole after the transplant surgery and were maintained with trimethoprim-sulfamethoxazole indefinitely. Preemptive therapy was used to prevent cytomegalovirus (CMV) disease for up to 9 mo of the planned duration of the multiple-target therapy.

### Outcomes of Interest and Definitions

CR, defined as a reduction of proteinuria to <0.3 g/d, and PR, defined as a 50% reduction of proteinuria compared with the baseline value, provided that the final value was <3.5 g/d, were assessed 12 mo after the start of treatment.

Safety endpoints were assessed at 12 mo from the start of treatment, including the need for treatment discontinuation, infectious complication, and recipient death.

Delayed graft function was defined as the need for at least 1 dialysis session during the first week after transplantation. Kidney allograft function was evaluated at diagnosis and at months 1, 3, 9, and 12 from the beginning of the treatment for posttransplant FSGS. The estimated glomerular filtration rate was calculated using the Chronic Kidney Disease Epidemiology Collaboration equation for patients older than 18 y and the *Revised Bedside Schwartz Formula* for patients younger than 18 y. Posttransplant FSGS was considered “immediate” if developed within 24 h of the onset of diureses after KT, “early” if developed after 24 h but within the first 12 wk, and “late” if it occurred after 12 wk from KT.^10^

### Statistical Analysis

Continuous variables were presented as mean and SD, or median, minimum, and maximum when applicable, and categorical variables were presented as absolute frequency and percentage. Statistical analysis was performed using SPSS version 23 software (IBM SPSS Statistics).

## RESULTS

Thirteen patients were diagnosed with posttransplant FSGS and received the multiple-target therapy. Of them, 5 (38.5%) had biopsy-confirmed primary FSGS in the native kidneys. No patient had undergone nephrectomy before transplantation. Two patients received an HLA identical living donor KT and 1 deceased donor retransplant. Nine patients received rabbit antithymocyte globulin induction therapy. Seven patients received tacrolimus and azathioprine or mycophenolate as maintenance and 4 patients were treated with tacrolimus and sirolimus (Table 1).

**TABLE 1.** Demographic characteristics of the study population

Parameters	Total (N = 13)
Recipient age, y, median (min–max)	43 (4–63)
Recipient sex, male, n (%)	10 (76.9)
Body mass index, kg/m^2^, mean ± SD	23 ± 4.5
Recipient race, n (%)	
Caucasian	4 (30.7)
Afro descendant	0 (0)
Asian	0 (0)
Mixed race	9 (69)
Cause of chronic kidney disease, n (%)	
bcFSGS	5 (38.5)
pFSGS	8 (61.5)
>250 mL residual urine output, n (%)	6 (46.1)
Serum albumin, g/dL, median (min–max)	3.3 (2.2–3.8)
<3 g/dL, n (%)	3 (23)
Age at diagnosis of CKD, median (min–max)	39 (3–60)
Time from CKD diagnosis to dialysis, mo, median (min–max)	59 (0–252)
Time on dialysis, mo, median (min–max)	18 (1–84)
HLA mismatches, median (min–max)	2 (0–3)
Donor age, y, median (min–max)	35 (14-59)
Donor source	
Living HLA identical	2 (15.4)
Deceased standard criteria	9 (69.2)
Deceased expanded criteria	2 (15.4)
Previous kidney transplant, n (%)	1 (7.7)
Induction therapy, n (%)	
rATG induction, n (%)	9 (69.2)
Basiliximab induction, n (%)	2 (15.4)
Maintenance immunosuppression, n (%)	
Cyclosporine, azathioprine, steroid	2 (15.4)
Tacrolimus, azathioprine, steroid	5 (38.4)
Tacrolimus, mycophenolate, steroid	2(15.4)
Tacrolimus, sirolimus, steroid	4 (30.8)

bcFSGS, biopsy-confirmed focal segmental glomerulosclerosis; CKD, chronic kidney disease; pFSGS, presumed FSGS; rATG, rabbit antithymocyte globulin.

### Multiple-target Therapy Parameters

The mean CsA whole blood trough concentrations were within the target therapeutic range from week 1 to 9 (Table 2). Mean prednisone daily doses were slightly lower from week 1 to 6, but within the protocol-defined doses thereafter.

**TABLE 2.** Multiple-target therapy characteristics

Parameters	Total (N = 13)
Time to treatment after diagnosis, d, median (min–max)	5 (0–34)
Cyclosporine concentration, ng/mL, mean ± SD	
Screening (n = 11)	141 ± 162
Week 1 (n = 12)	293 ± 145
Week 2 (n = 11)	374 ± 132
Week 3 (n = 10)	428 ± 226
Week 4 (n = 13)	279 ± 35.4
Week 5 (n = 13)	266 ± 98.2
Week 6 (n=13)	277 ± 94
Week 7 (n=10)	284 ± 187
Week 8 (n=12)	256 ± 102
Week 9 (n=11)	205 ± 94
Prednisone daily dose, mg/kg, mean ± SD	
Weeks 1–4 (n = 13)	0.85 ± 0.14
Weeks 5–6 (n = 13)	0.70 ± 0.20
Weeks 7–8 (n = 13)	0.51 ± 0.22
Weeks 9–10 (n = 12)	0.45 ± 0.30
Plasmapheresis sessions, n, median (min–max)	18 (2–66)
<10 sessions (n, %)	4 (30.7)
10–30 sessions	5 (38.4)
>30 sessions	4 (30.7)
Renin-angiotensin blockers, n (%)	6 (46)

Twelve patients started PP therapy within a maximum of 3 d after inclusion and 1 patient started after 66 d, postponed because of infectious complications. The patients received a median (min–max) of 18 sessions of PP (2–66; Table 2).

### Clinical Presentation, Efficacy, and Safety

All 5 patients with biopsy-confirmed FSGS in the native kidneys developed immediate or early posttransplant FSGS recurrence clinically manifested by delayed graft function or allograft dysfunction and nephrotic proteinuria (Table 3). The median time to diagnosis was 7 d and the median proteinuria was 7.2 g/g urinary protein/creatinine ratio. The allograft biopsies at diagnosis showed no evidence of segmental glomerulosclerotic lesions by light microscopy, and all immunofluorescence analyses were negative for immunoglobulins and complement deposits. No electronic microscopy analysis was obtained.

**TABLE 3.** Clinical characteristics and outcomes of the patients with posttransplant FSGS

Patients	Donor type	Age, y	Diagnosis	Time after transplant, d	Diagnosis			1 mo		3 mo		9 mo		12 mo		CMV Status	Treatment discontinuation, d	Outcome	Mean graft survival, mo
					Cr, mg/dL	U_P/C,_ g/g	Serum albumin, g/dL	Cr, mg/dL	U_P/C,_ g/g	Cr, mg/dL	U_P/C,_ g/g	Cr, mg/dL	U_P/C,_ g/g	Cr_,_ mg/dL	U_P/C,_ g/g	D/R			
1^*a*^	DD	4	DGF	4	3.38	29.79	3.2	1.28	57.3	0.7	50.0	0.61	15.32	0.69	14.6	+/+	–	NR	96 (F)
3^*a*^	DD	36	DGF	4	12.07	1.84	3.8	2.2	0.47	1.88	0.38	2.01	0.44	2.3	0.37	Unknown/+	48	PR	41
6^*a*^	DD	63	DGF	7	7.93	3.4	3.3	7.9	1.95	–	–	–	–	–	–	+/+	23	GL	3
12^*a*^	HLAI	64	Graft dysfunction	7	1.8	7.2	3.5	4.7	10.9	1.35	1.68	1.7	2.2	1.5	0.75	+/+	56	PR	87 (F)
13^*a*^	DD	5	DGF	12	2.38	20.4	3.5	1.7	22.8	1.03	26.2	–	–	–	–	Unknown/+	70	GL	8
8	HLAI	53	Graft dysfunction	26	2.02	15	3.3	1.1	0.5	1.2	0	2.2	0.29	3.1	0.74	+/+	–	CR	24
10	DD	48	bcFSGS	31	2.6	4.6	3.4	2.0	3.2	3.49	2.2	3.6	0.3	4.05	0.5	±	19	NR	61
7	DD	37	bcFSGS	68	3.9	6.3	3.7	3.93	4.8	5.5	3.5	–	–	–	–	+/+	7	GL	11
9	DD	43	bcFSGS	143	2.16	7.12	3.5	2.4	21	3.3	30	–	–	–	–	±	29	GL	10
11	DD	51	bcFSGS	195	1.6	4.8	2.5	1.2	2.6	1.5	4.1	2.6	7.1	2.5	6.7	±	90	NR	40
2	DD	61	bcFSGS	336	1.61	8.77	3.0	2.04	2.78	2.33	2.35	3.2	1.34	2.6	2	Unknown/+	75	NR	96 (F)
5	DD	40	bcFSGS	356	2.84	2.0	2.2	1.92	0.59	2.2	0.18	2.06	0	2.06	0	Unknown/+	–	CR	98 (F)
4	DD	54	bcFSGS	1432	1.8	2.8	2.3	1.33	1.75	1.83	0.41	–	–	–	–	±	27	GL	50

^*a*^Patients with biopsy-confirmed FSGS in the native kidney.

bcFSGS, biopsy-confirmed focal segmental glomerulosclerosis; CMV, cytomegalovirus; Cr, creatinine; CR, complete response; D, donor; DD, deceased donor; DGF, delayed graft function; (F), functioning allograft; GL, graft loss; NR, no response; PR, partial response; R, recipient; U_P/C_, urinary protein/creatinine ratio.

Three patients without biopsy-confirmed FSGS in the native kidneys also developed early posttransplant FSGS, with 2 of them already showing FSGS lesions in the allograft.

The remaining 5 patients without biopsy-confirmed FSGS in the native kidneys developed late posttransplant FSGS, with proteinuria and FSGS lesions in the allograft biopsies. Importantly, 4 of them were receiving sirolimus at the time of diagnosis but showed no improvement on a median of 42 d (11–168) after its discontinuation (Table 3).

Throughout the study, 10 patients had to be permanently discontinued from the protocol because of infectious adverse events (3 CMV disease, 3 CMV viremia, 3 herpes zoster, and 1 endocarditis). Of the patients who developed CMV infection, 2 were at high risk (seronegative recipients receiving an organ from a seropositive donor). As a secondary analysis, excluding 5 patients who had to definitively discontinue treatment within the first month, there were 25% of PRs (n = 2), 25% of CRs (n = 2), 25% of no remissions (n = 2), and 25% of graft losses (n = 2).

At 12 mo, global analysis showed 2 PRs, 2 CRs, 4 no remissions, and 5 allografts lost because of FSGS. There was no death during the follow-up. The mean graft survival at 8 y was 48 ± 36.4 mo (Table 3).

## DISCUSSION

In this prospective interventional study, the effectiveness of the multiple-target therapy for posttransplant FSGS could not be substantiated. This was attributed to the premature discontinuation of treatment in most patients because of infectious complications.

To date, no established standard of care therapy exists for the treatment of recurrence of FSGS. Noteworthy, Canaud et al^9^ achieved a remarkable CR rate in 90% of the patients, with no reported infectious adverse events. This outcome was achieved with the implementation of a multiple-target therapy addressing systemic immune dysregulation and podocyte cytoskeleton.^9^ Nevertheless, these remarkable results have not been replicated by other groups. In addition to our study, Lanaret et al^11^ also used this multiple-target therapy, showing that 27.5% of 109 patients failed to attain remission. Finally, in a comprehensive international multicenter retrospective study on FSGS recurrence, including 75 adult patients, multiple-target therapy was particularly absent as a treatment option (n = 3).

There are several factors associated with somewhat contrasting results. First, the definitive diagnosis of recurrent FSGS is challenging. The first 8 patients included 5 with biopsy-confirmed FSGS in the native kidneys and 3 with a history of nephrotic syndrome resembling those described by Canaud et al. The early onset and the nephrotic range proteinuria suggest that a circulating permeability factor probably causes podocyte damage.^12^ In this set of patients, proteinuria in the nephrotic range is the hallmark of the disease, although typical histopathological lesions only appear on light microscopy 4–6 wk after transplantation.^13,14^ In this subgroup of 8 patients, 1 showed complete and 2 PRs, but 6 patients discontinued treatment because of infectious adverse events.

Infectious complications are a real concern when dealing with posttransplant FSGS. In our previously published report, 70% of patients experienced infections. Lanaret et al^11^ also reported infectious adverse events in 79.5% of the patients. The high incidence of CMV infection is associated with the use of preemptive therapy and may be prevented during the course of the multiple-target therapy by considering pharmacological prophylaxis against CMV, such as those used by Canaud et al.

The last 5 patients, including 4 who received mammalian target of rapamycin inhibitor (mTORi), comprise a heterogeneous group showing predominantly late disease presentation. The previous use of mTORi raises the possibility of secondary FSGS. Although proteinuria has been associated with the use of mTORi, it usually occurs at low, nonnephrotic ranges and without histological lesions typical of FSGS.^15,16^ The lack of clinical and laboratory improvement after mTORi treatment discontinuation, with persistent nephrotic range proteinuria, prompted treatment.

Altogether, despite several limitations, our data confirm and highlight the complexity of the diagnosis and treatment of posttransplant FSGS. The lack of electron microscopy is the major limitation and valuable insights into the pathologic characteristics of FSGS were not elucidated. In addition, regional and ethnic demographic characteristics, endemic infectious diseases, and propensity to infections interfere directly with the efficacy of any immunosuppressive treatment, including the multiple-target therapy.

In conclusion, in this single-center prospective study, the efficacy of multiple-target therapy was limited by its toxicity, mainly represented by CMV infection. Future studies involving multicenter collaborations, randomized control trials with larger patient populations, and additional prophylaxis against infections are needed to identify effective and safe strategies to treat this complex disease.
